# Phenotypic characteristic of *Staphylococcus aureus* from subclinical mastitis in Etawah-crossbreed goats in Yogyakarta, Indonesia

**DOI:** 10.14202/vetworld.2022.2587-2592

**Published:** 2022-11-15

**Authors:** Widodo Suwito, Widagdo Sri Nugroho, Rahmat Setya Adji, Andriani Andriani, Eny Kusumaningtyas, Tri Martini

**Affiliations:** 1 Research Center Processing Food Technology, National Research Innovation Innovation Agency (BRIN), Gunungkidul 55861, Yogyakarta, Indonesia; 2 Department of Veterinary Public Health, Faculty of Veterinary Medicine, Universitas Gadjah Mada, Sleman 55281, Yogyakarta, Indonesia; 3 Veterinary Research Center, National Research Innovation Agency (BRIN), Bogor 16124, West Java, Indonesia; 4 Research Center Sustainable Production System and Life Cycle Assessment, Serpong 15311, Banten, Indonesia

**Keywords:** Etawah-grade goats, phenotypic, *Staphylococcus aureus*, subclinical mastitis

## Abstract

**Background and Aim:**

Subclinical mastitis (SCM) in Etawah-grade (PE) goats in Yogyakarta, Indonesia, is commonly due to *Staphylococcus aureus*. At present, *S. aureus* from SCM in PE goats in Yogyakarta has not been characterized. Therefore, this study aimed to phenotypically characterize *S. aureus*, which has been isolated from SCM of PE goats.

**Materials and Methods:**

A total of 314 lactating PE goats were collected from 60 PE goat farms (e.g., Sleman, Bantul, and Kulonprogo) located in parts of Yogyakarta with an average age of 3–4 years old, three of which showed SCM based on the California mastitis test (CMT). Subclinical mastitis is confirmed in PE goats if CMT shows ++ or +++. Furthermore, *S. aureus* was detected by biochemical assays. *Staphylococcus aureus* could determine hemolysin (Hae), coagulase (Coa), clumping factor (Cf), and antibiotic susceptibility. Hemolytic bacteria were detected by culturing on blood agar plate, and Cf was detected by slide agglutination. The production of Coa was detected by tube coagulation. *Staphylococcus aureus* susceptibility was determined by antimicrobial agar diffusion using a paper disc.

**Results:**

Phenotypically characterized *S. aureus* from PE goats with SCM in Yogyakarta, Indonesia, Coa−, Cf−, and Hae− were found to be resistant to erythromycin (ERYTHRO), ampicillin (AMP), penicillin (PEN-G), and sulfamethoxazole (SULFA).

**Conclusion:**

The phenotypic characteristic of *S. aureus*, which was obtained from SCM in PE goats in Yogyakarta, consists of Coa, and Cf−. *S. aureus* cannot perform hemolysis of red blood cells. This phenotypic characteristic can prevent and control SCM in PE goats. Several antibiotics such as ERYTHRO, AMP, PEN-G, and SULFA were no longer effective for treating SCM in PE goats because *S. aureus* has developed its resistance to these antibiotics.

## Introduction

Etawah-grade (PE) goat is a breed of goat that can produce milk, which is commonly found in Yogyakarta, Indonesia, particularly in Sleman, Bantul, and Kulonprogo districts. Subclinical mastitis (SCM) is a prevalent disease in milk production because it reduces milk production and alters milk composition [1, 2]. SCM in PE goat causes many disadvantages as it reduces goat milk production to 37%–60% [3]. PE goat farmers in Yogyakarta, Indonesia, do not have a comprehensive knowledge of SCM in PE goats because this disease is asymptomatic. Suwito *et al*. [4] reported that 35% of SCM cases in PE goats were due to *Staphylococcus aureus*. The number of somatic cells in milk is used for detecting SCM in goats, but it has no practical use in the field. The California mastitis test (CMT) is a reagent commonly used to detect SCM on farms because of its simplicity. The principles of the CMT test can be used indirectly to measure somatic cell counts (SCC) in milk [5]. Subclinical mastitis in PE goats can be easily detected by the CMT test; however, the category of standard for goats and cows is different. The detection of SCM in PE goats can be more easily performed using the CMT test. Physiologically, goat milk has a higher SCC than cow milk [6]. Therefore, the SCM criteria for goats and cows are different because goat milk secretes more apocrine, whereas cow milk secretes more merocrine [6]. A goat is considered to have SCM if the CMT shows ++ or positive +++ [7].

*Staphylococcus* spp. have been reported to be a common pathogen causing SCM in dairy goats [8]. Generally, *S. aureus* is a more common cause of SCM in goats than in other staphylococci [9]. *Staphylococcus aureus* has several characteristics, such as being Gram-positive, facultative aerobic, non-sporulating, non-motile, catalase-positive, and oxidase-negative. Furthermore, it can be found on the surface of human, and animal skin [10]. *Staphylococcus aureus* in PE goat with SCM has different virulence factors. Differences in *S. aureus* virulence factors also have profound implications for infection mechanisms. Hemolysin (Hae) is an important virulence factor mechanism of red blood cell (RBC) lysis in *S. aureus* [10]. Non-hemolytic *S. aureus* is less pathogenic, but it has other virulence factors that are important to the infection mechanism of the sample, such as coagulase (Coa).

Subclinical mastitis in goats is due to *S. aureus* hemolyzed with Coa-positive (Coa+) or Coa-negative (Coa−) [11]. In Yogyakarta, Indonesia, most cases of SCM found in PE goats are due to *S. aureus*; however, they have not been characterized, for example, Hae, aggregation factor (Cf), Coa, and antimicrobial susceptibility patterns.

Thus, this study aimed to explain in detail the phenotypic characteristics of *S. aureus*, which has been isolated from PE goats with SCM in Yogyakarta, Indonesia.

## Materials and Methods

### Ethical approval

This study was approved by the Local Ethics Committee for Animal Experiments, Faculty of Veterinary Medicine, Universitas Gadjah Mada, Yogyakarta, Indonesia (Approval no: 00081/EC-/FKH/Eks./2021).

### Study period and location

The study was conducted from March to August 2021 at Department of Veterinary Public Health, Faculty of Veterinary Medicine, Universitas Gadjah Mada, Yogyakarta, Indonesia,

### Animals

The sample size of lactating PE goats was calculated using the following formula: n = 4PQ/L^2^ [12]. The 95% confidence level and 5% gallat were desired using the apparent prevalence (P) of SCM in PE goats (12%) [13]. A total of 314 lactating PE goats were collected from 60 PE goat farms in different parts of Yogyakarta (such as Sleman, Bantul, and Kulonprogo), and SCM was tested using a CMT reagent. The CMT test was performed on PE goat farms. The isolation, identification, and characterization of *S. aureus* were performed in the Laboratory of Veterinary Public Health, Faculty of Veterinary Medicine, Universitas Gadjah Mada, Indonesia.

### Determination of SCM in PE goats

Subclinical mastitis in PE goats was determined using a CMT reagent. A CMT test was performed by mixing 3–4 mL of PE goat milk and a CMT reagent of the same amount and stirring them immediately in a circular motion. Gel formation showed that SCM occurred in PE goats. The score obtained from the CMT test was divided into four grades based on the degree of response consisting non (0) and positive (+, +++, and ++++). In PE goats, non (0) and positive (+) CMT scores can be classified as negative SCM, whereas positive (++ and +++) CMT scores indicate SCM [7].

### Collection of samples

Samples were collected aseptically from PE goats. The teats of PE goats were wiped with a cotton swab soaked in 70% alcohol, and then some of the milk streams were discarded. Up to 10–15 mL of PE goat milk was collected in sterile test tubes and immediately sent to the Laboratory of Veterinary Public Health, Faculty of Veterinary Medicine, Universitas Gadjah Mada, Indonesia. The PE goat milk samples were kept at 4°C before the isolation and identification of *S. aureus*.

### Detection of *S. aureus*

*Staphylococcus aureus* was detected in accordance with the Bacteriological Analysis Manual method [14]. Milk SCM samples from PE goats were added to 25 mL of buffered peptone water (CM: 0509, Oxoid Ltd., Basingstoke, United Kingdom), incubated at 37°C for 24 h, and then cultured on mannitol salt agar (MSA) (CM: 0085 Oxoid Ltd.). Afterward, the samples were again incubated at 37°C for 24 h. Colonies grown on yellow MSA medium had a cocci shape after being stained with purple gram, which were then biochemically tested to identify *S. aureus*.

### Phenotypic characterization

Phenotypic features of *S. aureus* included Hae, Cf, and Coa [10]. Hemolytic *S. aureus* was tested by culturing on blood agar plates (BAP) (CM: 0055B; Oxoid Ltd.) and incubating at 37°C for 24 h. Hemolytic *S. aureus* was divided into three groups, including alpha, beta, and non-hemolytic [10]. The catalase assay was performed by aseptically collecting *S. aureus* colonies using *Ose*. Next, the colony was placed on the object glass and added with one drop of 3% H_2_O_2_. Catalase was considered as positive if bubbling O_2_ gas was present [10]. The clumping factor (Cf) was detected by slide agglutination, wherein one colony of *S. aureus* was collected from BAP (CM: 0055B; Oxoid Ltd.) using a sterile *Ose*. Then, the colonies were placed on the object glass and mixed with sterile water until a homogeneous suspension was obtained. Two drops of rabbit plasma were added to the *S. aureus* suspension, and the mixture was aseptically stirred using *Ose* and observed for possible agglutination. The tube coagulation assay was used to detect Coa in *S. aureus* [10]. A 24-h culture of *S. aureus* in 5-mL brain heart infusion (CM:1135, Oxoid Ltd.) was mixed with rabbit plasma in the same proportion. This mixture was immersed in a water bath containing 37°C water, and gel formation was observed after 15 and 30 min.

### Antibiotic susceptibility testing

Antibiotic susceptibility testing was performed using the diffusion agar method on mueller–hinton agar (CM 0337, Oxoid Ltd.) recommended by Clinical and Laboratory Standards Institute [15]. Antibiotics tested included ampicillin (AMP) (10 μg), cefoxitin (30 μg), erythromycin (ERYTHRO) (15 μg), gentamicin (10 μg), neomycin (30 μg), oxacillin (5 μg), oxytetracycline (30 μg), penicillin (PEN-G) (10 IU), sulfamethoxazole (SULFA) (300 μg), and tetracycline (30 μg). Plates were incubated at 37°C for 24 h, and sensitivity patterns were observed and classified as sensitive, intermediate, or resistant based on the diameter of the inhibition zone [15].

### Statistical analysis

Microsoft Excel was used to classify and code data describing the active use of CMT assays, isolation, and identification, *S. aureus*, phenotypic characterization, and antibiotic susceptibility testing samples. Descriptive statistics were used to analyze the total number of positive CMT, isolates of *S. aureus*, phenotypic characteristics, and antibiotic susceptibility.

## Results

Subclinical mastitis in PE goats occurs if the CMT test shows positive ≥++. The determination of SCM in PE goats from Yogyakarta, Indonesia, based on the CMT test is presented in Table-1. In Sleman, the number of *S. aureus* isolates was higher than in Bantul and Kulonprogo districts. Isolation and identification of *S. aureus* from SCM in PE goats are presented in Table-2. The phenotypic characteristics of *S. aureus* include Coa, Cf, and hemolysis. The phenotypic characteristics of *S. aureus* in PE goats with SCM in Yogyakarta, Indonesia, are shown in Table-3. Furthermore, the susceptibility of *S. aureus* in PE goats with SCM in Yogyakarta, Indonesia, is shown in Table-4.

**Table-1:** Determination of SCM in PE goats based on CMT in Yogyakarta.

District	Total of PE goat	CMT assay	

		(-)	(≥ ++)
Sleman	282	198	84
Bantul	65	47	18
Kulonprogo	37	27	10
Total	384	272	112

-=Negative SCM; ( ≥ ++)=Positive SCM, SCM=Subclinical mastitis, PE=Etawah-grade, CMT=California mastitis test

**Table-2:** Detection *S. aureus* from SCM in PE goats in Yogyakarta.

District	Total PE goats SCM	Isolate *S. aureus*
Sleman	84	7
Bantul	18	6
Kulonprogo	10	7
Total	112	28

SCM=Subclinical mastitis, PE=Etawah-grade, *S. aureus*=*Staphylococcus aureus*

**Table-3:** Characterization of *S. aureus* from SCM in PE goats at Yogyakarta.

District	Coa+	Coa-	Cf+	Cf-	Hae+	Hae-
Sleman (n=7)	2	5	2	5	1	6
Bantul (n=6)	3	3	1	5	0	6
Kulonprogo (n=7)	3	4	3	4	0	7
Total (n=20)	8	12	6	14	1	19

Coa=Coagulase, Cf=Clumping factor, Hae=Hemolytic, SCM=Subclinical mastitis, PE=Etawah-grade, *S. aureus=Staphylococcus aureus*

**Table-4:** Susceptibility of antibiotics against *S. aureus* from SCM in PE goats in Yogyakarta.

Antibiotic	Sleman			Bantul			Kulonprogo		
		
	(n = 7)			(n = 6)			(n = 7)		
		
	S (%)	I (%)	R (%)	S (%)	I (%)	R (%)	S (%)	I (%)	R (%)
AMP (10 µg)	5	0	95	25	5	70	20	5	75
CEFOX (30 µg)	100	0	0	100	0	0	100	0	0
ERYTHRO (15 µg)	10	5	85	5	10	85	20	0	80
GENTA (10 µg)	85	15	0	90	10	0	85	15	0
NEO (30 µg)	75	25	0	85	15	0	85	15	0
OXA (5 µg)	100	0	0	100	0	0	100	0	0
OXY (30 µg)	100	0	0	100	0	0	100	0	0
PEN- G (10IU)	10	0	90	30	0	70	40	0	60
SULFA (300 µg)	20	0	80	40	0	60	30	0	70
TETRA (30 µg)	90	5	5	100	0	0	100	0	0

AMP=Ampicillin, CEFOX=Cefoxitin, ERYTHRO=Erythromycin, GENTA=Gentamycine, NEO=Neomycine, OXA=Oxacillin, OXY=Oxytetracycline, PEN-G=Penicillin, SULFA=Sulfamethoxazole, TETRA=Tetracycline, S=Sensitive, I=Intermediate, R=Resistant, SCM=Subclinical mastitis, PE=Etawah-grade, *S. aureus=Staphylococcus aureus*

## Discussion

In Yogyakarta, Indonesia, PE goat is a breed of goat that yields milk. However, SCM in PE goats can be perceived as an economical disease that decreases the milk yield of goats. Most PE goat farmers in Yogyakarta, Indonesia, do not have adequate knowledge on the possibility of SCM in PE goats. Consequently, it inflicts a financial loss on PE goat farmers. In general, SCM in PE goats can be detected by a CMT test, and farmers cannot test their PE goats because most PE goat farms in Yogyakarta, Indonesia, are small farms that own 3–6 goats. Additionally, the CMT reagent is expensive; thus, a CMT test cannot be performed. This research shows that the prevalence of SCM in PE goats in Yogyakarta, Indonesia, is 29.29%. Some researchers have reported that the prevalence level of SCM in goats is 37.19%, 38%, 45.82%, and 50% [16–19]. Moreover, the prevalence level of SCM in PE goats in Yogyakarta, Indonesia, is lower than that in other countries. Various factors greatly affect the prevalence level of SCM in goats, one of which is milk yield. High milk yield is a risk factor influencing the occurrence of SCM in goats. In goats, SCM can be affected by various factors such as high milk yield, lactation age >4 years, litter size >2, weaning age, body condition score, and end of lactation [1, 4].

Bacterial infection is a common cause of clinical mastitis (CM) and SCM in PE goats, of which *S. aureus* is the major pathogen [4]. The prevalence of *S. aureus*, which causes SCM in Yogyakarta, Indonesia, reached 25%. The prevalence of *S. aureus* in SCM in Yogyakarta, Indonesia, is lower than that found in other reports, reaching 36.84% [17]. Therefore, the prevalence of *S. aureus* in goats with SCM in Yogyakarta, Indonesia, remains low. Differences in the prevalence of *S. aureus* in goats with SCM can be affected by the milking management factor. Traditional milking by hand can cause SCM because of *S. aureus* compared with modern milking by machine because hands are the source of *S. aureus* that causes SCM in goats [20].

A virulence factor plays an important role in the pathogenesis of *S. aureus* in PE goats with SCM. Various virulence genes in *S. aureus* encode a wide range of virulence factors such as toxic shock syndrome toxin-1 (TSST-1); enterotoxins; and Hae, Coa, Cf, and Panton-Valentine leukocidin [10]. *Staphylococcus aureus*, which causes SCM in PE goats in Yogyakarta, Indonesia, has several major characteristics such as Coa−, Cf−, and Hae−. Hemolytic *S. aureus* with alpha type is the only isolate that was identified from SCM found in PE goats in Yogyakarta, Indonesia. Only a small number of Hae virulence factors in *S. aureus* are found in PE goats with SCM, but another virulence factor such as enterotoxin or TSST-1 is observed. Commonly, *S. aureus* Hae is identified in CM than in SCM in goats and cattle, and alpha-Hae toxin is the most prevalent virulence factor in *S. aureus* [21].

Subclinical mastitis observed in PE goats in Yogyakarta, Indonesia, shows that coagulase-negative *staphylococci* (CoNS) is higher than coagulase-positive *staphylococci*. This finding is consistent with other studies, which have concluded that CoNS is the causative agent of SCM cases in dairy goats [22–24]. Coagulase-negative *staphylococci* is commonly detected in goat milk, and these microorganisms can frequently cause SCM infections that persist for several months, even during the dry period [25].

*Staphylococcus aureus* isolated from PE goat SCM produces Cf and Coa. These enzymes are required for the pathogenesis of SCM in PE goats. Coagulase plays an important role in infection endocarditis caused by *S. aureus* because it facilitates the bonding of a procoagulant with fibrinogen [10]. In *S. aureus* isolates, Cf, and Coa are similar, although they have different roles in pathogenesis. In the pathogenesis of *S. aureus*, Cf contributes to the bonding with protein A [10].

Antibiotic susceptibility testing should be performed before the prescription of antimicrobials to avoid excessive and unnecessary usage of antibiotics. In this study, we have tested most of the commonly used antimicrobials against the clinical isolates of *S. aureus* to understand the level of drug resistance in the isolated pathogens. In addition, the resistance of *S. aureus* to PEN-G is 60%–90%, which is higher than that in other countries, as it has been reported that such resistance reaches 12% in Italy [26]. Other researchers have reported that the resistance of *S. aureus* in goats with CM and SCM in Malaysia to PEN-G reached 22% [27]. This shows that the prevalence of antibiotic resistance in other countries is affected by some factors such as farm management.

In Yogyakarta, Indonesia, particularly in the Sleman district, antibiotic is freely used in PE goat farms because of its accessibility in the market, and it can be bought without a veterinary prescription. Consequently, PE goat farmers use antibiotics without any veterinary consent, thereby resulting in mutation. Penicillin can destroy the cell wall of Gram-positive bacteria [28]. It is a commonly used antibiotic in PE goat farms in the Sleman district. Penicillin has various derivatives, one of which is AMP. It is also having a ring beta-lactam structure [28]. Resistance to PEN-G and AMP, which is due to *S. aureus* from SCM in PE goats, produces a beta-lactamase enzyme. The beta-lactamase enzyme will destroy the ring beta-lactam, resulting in the ineffectiveness of PEN-G [28].

Erythromycin resistance occurs in *S. aureus* isolated from PE goats with SCM in Yogyakarta, Indonesia, reaching 80%–85%. This study supports the finding that resistance of *S. aureus* from goats in Jordan accounts for 70%–80% [29]. The high prevalence of ERYTHR Oresistance in *S. aureus* from SCM found in PE goats results from several factors. Etawah-grade goat farmers frequently use antibiotics without proper dosage, and this habit potentially arises the possibility of microbial mutation. Therefore, genetic mutation is a leading factor in antibiotic resistance.

In general, ERYTHRO resistance reduces antibiotic affinity to bacteria [30]. This occurs through several mechanisms such as reflux systems, methylation, or inactivated enzymes, as well as gene mutations in ribosomal proteins and the 23S rRNA gene. Antibiotic affinity can decrease the enzymatic detoxification of antibiotics or bacteria modified [30]. In addition, the access of drugs into bacterial cells has been lost because of active reflux or the decrease of entry drugs into molecular protein bacteria. Moreover, the presence of chromosomal mutations can change the location of the ERYTHRO bond in 23S rRNA [30].

SULFA is an antimicrobial group sulfa that has a broad-spectrum mechanism [30]. Resistance of *S. aureus* from SCM in PE goats from Yogyakarta, Indonesia, to SULFA accounts for 60%–80%. In this study, resistance to SULFA was a higher degree than *S. aureus* mastitis in Nigeria and Italy, which account for only 25% [31, 32]. In Yogyakarta, Indonesia, SCM is rarely treated with SULFA despite high occurrence resistance. Penicillin G is commonly used by veterinarians to treat CM in PE goats in Yogyakarta, Indonesia. Thus, the case of *S. aureus* resistance to SULFA remains high, although it is rarely used. It occurs because of gene mutation. Some literature states that *S. aureus* resistance to SULFA is mediated by the following five mechanisms: Permeability barrier and or efflux pumps, naturally insensitive enzymatic target, regulatory changes in the enzymatic target, change in mutational or recombinational enzymatic target, and acquired resistance by drug-resistant enzymatic targets.

## Conclusion

The phenotypic characteristic of *S. aureus*, which was obtained from SCM in PE goats in Yogyakarta, consists of Coa and Cf−. *Staphylococcus aureus* cannot perform hemolysis of RBCs. This phenotypic characteristic can prevent and control SCM in PE goats. Several antibiotics such as ERYTHRO, AMP, PEN-G, and SULFA were no longer effective for treating SCM in PE goats because *S. aureus* has developed its resistance to these antibiotics.

## Authors’ Contributions

WS, WSN, AA, RSA, EK and TM: Performed the study (samples collection, milk test processing, designed the experiment, and analyzed the data). WS, WSN, and TM: Carried out SCM test. EK, RSA, and AA: Sensitivity antibiotic test and phenotypic characteristic. All authors have read and approved the final manuscript.
